# Cases of Creutzfeldt-Jakob disease in young individuals: open questions regarding aetiology

**DOI:** 10.3389/fncel.2025.1571662

**Published:** 2025-04-14

**Authors:** Ilia V. Baskakov

**Affiliations:** ^1^Center for Biomedical Engineering and Technology, University of Maryland School of Medicine, Baltimore, MD, United States; ^2^Department of Neurobiology, University of Maryland School of Medicine, Baltimore, MD, United States

**Keywords:** prion, chronic wasting disease (CWD), BSE (mad cow disease), Creutzfeldt-Jakob disease (CJD), cross-species transmission

Recently, two young individuals, aged 15 and 21, were diagnosed with sporadic Creutzfeldt-Jakob disease (sCJD) in Canada and the United States, respectively (D'Arcy et al., 2019; Ahn et al., 2024). Considering that the mean age of onset for sCJD is 67 years, these early onset cases are exceedingly rare. Both patients were methionine/valine heterozygous at codon 129 of the prion protein gene (PRNP) and were classified as the MV1 subtype of sCJD. Both individuals exhibited an atypical clinical presentation and unusual profile of PrP^Sc^, notably lacking the diglycosylated glycoform. Following clinical onset, disease progression was remarkably slow in both cases, with disease durations of 119 and 39 months in the 15- and 21-year-old individuals, respectively.

Although sCJD in adolescents is very rare, such cases are not unprecedented. Two adolescent cases, in which patients succumbed to the disease at ages 16 and 20, were previously reported in the United Kingdom (Murray et al., 2008). In one of these cases, the diagnosis of sCJD was confirmed based on neuropathological findings, PrP^Sc^ subtype analysis, and transmission in mice. Additionally, a 19-year-old patient was identified in Germany among a cohort of 52 sCJD patients aged 50 or younger at symptom onset during 1993–2003 (Boesenberg et al., 2005). The clinical manifestations in younger sCJD patients differ from those in older individuals in terms of clinical signs, disease duration, and neuropathological lesion profiles (Pocchiari et al., 2004; Boesenberg et al., 2005), with a younger age at onset correlating with prolonged survival (Pocchiari et al., 2004). In a cohort of 2,304 sCJD cases identified in Western Europe between 1993 and 2000, two cases with onset between 11 and 20 years exhibited disease durations of 54 and 58 months, respectively (Pocchiari et al., 2004). Valine homozygosity at codon 129 was more frequent in the “young” cohort (patients younger than 50 at symptom onset) compared to the “old” cohort (patients older than 50; Boesenberg et al., 2005). The ratio of type 1 to type 2 PrP^Sc^ subtypes, determined based on glycoform ratios and the electrophoretic mobility of proteinase K-resistant core, did not differ between the two groups (Boesenberg et al., 2005).

In the case of the 21-year-old patient identified in the United States (Ahn et al., 2024), the absence of the diglycosylated isoform and the higher molecular weight of the mono- and unglycosylated isoforms, relative to those typically observed in type 1 or type 2 PrP^Sc^ subtypes, raise questions about whether the disease phenotype aligns with any known sCJD subtype. The possibility of CJD transmission was ruled out, as the patient had never undergone any medical procedures associated with a risk of prion transmission nor traveled to countries affected by bovine spongiform encephalopathy (BSE; Ahn et al., 2024). Nevertheless, although such an early age of onset is rare for sCJD, it is characteristic of variant CJD (vCJD), which has a median onset age of 28 years.

Unlike sCJD, vCJD is acquired through the consumption of beef or beef products contaminated with BSE, also known as mad cow disease—a fatal prion disorder in cattle (Prusiner, 1997). While prion diseases primarily affect the brain, the lymphoreticular system playing a crucial role in transmission of BSE to humans (Hilton et al., 2004b; Aguzzi et al., 2013). Shortly after exposure, lymphotropic prion strains, including BSE, colonize secondary lymphoid organs (SLOs), where they exploit follicular dendritic cells to replicate and accumulate before spreading to the central nervous system (CNS; Hilton et al., 1998; Brown et al., 1999; McCulloch et al., 2011; Mabbott, 2012; Aguzzi et al., 2013). Similar to BSE, chronic wasting disease (CWD), a prion disease affecting cervids, also exhibits strong lymphotropism (Sigurdson et al., 1999).

CWD, which affects deer, elk, and moose, has been rapidly expanding across Canada and the U.S. As of early 2025, CWD has been detected in 36 U.S. states (USGS, 2025). The disease is highly contagious and primarily transmitted horizontally among cervids. CWD prions are shed in bodily fluids such as urine, saliva, and feces, contributing to persistent environmental contamination, particularly in soil (Tamgüney et al., 2009; Bartelt-Hunt and Bartz, 2013; Henderson et al., 2015, 2017; Davenport et al., 2018; Denkers et al., 2020; Tennant et al., 2020; Hwang et al., 2021; Denkers et al., 2024; Kuznetsova et al., 2024). Prions can be taken up by plants from contaminated soil and accumulate at levels sufficient for transmission to animals (Pritzkow et al., 2015; Carlson et al., 2023). Additionally, ticks have been shown to carry lethal doses of CWD infectivity (Inzalaco et al., 2023). While a substantial portion of the U.S. and Canadian populations is exposed to CWD through environmental contamination, the risk of transmission to humans is considered very low due to a significant species barrier.

The species barrier of CWD transmission to humans has been extensively studied using mouse models expressing human prion protein (PrP^C^; Kong et al., 2005; Sandberg et al., 2010; Wilson et al., 2012; Race et al., 2019; Hannaoui et al., 2022; Race et al., 2022; Wadsworth et al., 2022). In nearly all studies, humanized mice inoculated with CWD prions showed no clinical or subclinical disease and no detectable prion infectivity, with one notable exception (Hannaoui et al., 2022). In that study, infected humanized mice exhibited atypical clinical signs, prion seeding activity, and transmissible prion infectivity (Hannaoui et al., 2022).

In all previous studies assessing this risk, the intracranial (ic) route was used to administer CWD prions to humanized mice (Kong et al., 2005; Sandberg et al., 2010; Wilson et al., 2012; Race et al., 2019; Hannaoui et al., 2022; Race et al., 2022; Wadsworth et al., 2022). Ic inoculation is the most effective method for transmitting prions both within and across species (Race et al., 2009). However, successful cross-species transmission of lymphotropic prion strains appears to depend on SLOs for adaptation to a new host. Among the aforementioned studies, only one examined PrP^Sc^ accumulation in the spleen following CWD transmission to humanized mice (Wilson et al., 2012). When prions, such as BSE and CWD, cross species barriers, SLOs consistently exhibit greater permissiveness to prion replication than the brain (Béringue et al., 2012). Moreover, even after ic inoculation, lymphoreticular tissues exhibit a higher capacity than the brain to sustain and replicate lymphotropic prion strains, particularly at low-dose exposure (Halliez et al., 2014). This may be attributed to differences in glycosylation and sialylation between prions residing in the lymphoreticular system and those in the brain (Srivastava et al., 2015; Wagner et al., 2022). In spleens and lymph nodes, prions exhibit increased sialylation, potentially enhancing their resistance to clearance by innate immune system (Srivastava et al., 2015, 2017). Consequently, SLOs may provide a more favorable environment than the brain for prion adaptation to a new species. Furthermore, prion isolates, including CWD, have been shown to produce divergent disease phenotypes when introduced via ic versus peripheral routes, suggesting that brain and lymphoreticular tissues preferentially support different variants of PrP^Sc^ present in natural prion isolates (Béringue et al., 2012; DeFranco et al., 2024). Whether peripheral exposure facilitates more efficient cross-species adaptation of CWD prions remains unclear. Nonetheless, assessing PrP^Sc^ accumulation in SLOs following ic inoculation of humanized mice could provide a more sensitive approach for evaluating the potential risk of CWD transmission across species.

Additionally, with one exception (Wilson et al., 2012), all previous studies assessing the zoonotic potential of CWD have employed humanized mice homozygous for either 129MM or 129VV PrP (Kong et al., 2005; Sandberg et al., 2010; Race et al., 2019; Hannaoui et al., 2022; Race et al., 2022; Wadsworth et al., 2022). In the study that employed heterozygous 129MV humanized mice, the risk of transmission was assessed using only one CWD isolate (Wilson et al., 2012). In 129MV hosts, PrP^Sc^ structures must accommodate both 129M and 129V PrP molecules, likely resulting in an alternating incorporation of these isoforms. The presence of both 129M and 129V PrP^C^ substrates is expected to boost the conformational diversity of PrP^Sc^ variants. Whether the structure of 129MV PrP^Sc^ is more compatible with CWD strains than that of 129MM or 129VV PrP^Sc^, and whether the 129MV genotype is more susceptible to CWD prions, remains to be investigated.

With the continuous geographical expansion of CWD into highly populated areas and its increasing prevalence, human exposure—including that of children—to high doses of CWD prions via the environment may become unavoidable. The decomposition of carcasses from free-ranging deer infected with CWD could create environmental hotspots containing high concentrations of prions, posing long-term risks to ecosystems.

The uptake of prions by plants raises the possibility of contamination in the food chain, including dairy products. Evidence from prion research suggests that prions can be present in the mammary glands and milk of sheep incubating scrapie, the prion disease of sheep (Ligios et al., 2005; Lacroux et al., 2008; Maddison et al., 2009). If CWD prions can be adsorbed by the digestive system of cattle without causing clinical disease, they may still be excreted into milk, thereby introducing an unrecognized route of human exposure. The potential for milk contamination in dairy cattle that are not infected but are persistently exposed to CWD prions in contaminated environments warrants investigation.

A number of species, including goats, sheep, swine, rodents, mink, ferrets, raccoons, and possibly wild pigs and cattle are susceptible to CWD (Hamir et al., 2005, 2006; Raymond Gregory et al., 2007; Sigurdson et al., 2008; Heisey et al., 2010; Greenlee et al., 2012; Kurt and Sigurdson, 2016; Moore et al., 2017, 2019, 2022; Soto et al., 2025). Prions are subject to evolution and adaptation (Li et al., 2010; Baskakov, 2014). Upon transmission to new hosts, prion replication in a novel molecular environment enhances the conformational diversity of PrP^Sc^ variants, accelerating evolution and generating new strains with altered transmission characteristics (Gonzalez-Montalban et al., 2013; Makarava and Baskakov, 2013; Katorcha et al., 2018). As such, interspecies passage of CWD through different hosts may serve as a breeding ground for novel prion strains to emerge.

To date, more than 10 distinct CWD strains have been identified in deer, elk, moose, and reindeer (Otero et al., 2023; Sun et al., 2023). Assessing the transmissibility of diverse CWD strains is crucial for evaluating the potential risk of transmission to humans. Due to prion protein gene polymorphisms, cross-species transmission of CWD strains among different cervid species can alter strain properties, potentially leading to the emergence of novel variants with modified transmission characteristics (Bian et al., 2019, 2021; Otero et al., 2023). Such adaptations could expand the range of hosts susceptible to CWD.

Given the scale of potential CWD exposure, rare instances of transmission to humans might be expected beyond those directly linked to hunting. Since the clinical presentation of CWD in humans has not been defined, it is challenging to determine whether individuals diagnosed with sporadic sCJD at young ages, such as 15 and 21 years old, may have been infected with CWD. Autopsy of SLOs, including the spleen, lymph nodes, and tonsils, could help differentiate between sporadic and acquired forms of CJD. In sCJD patients, prions are detected in SLOs at a low prevalence (Glatzel et al., 2003), whereas in vCJD cases, linked to consumption of BSE-contaminated products, prions have been found in lymphoreticular tissues at a 100% rate (Hill et al., 1999; Ironside et al., 2002). Similarly, if CWD were transmissible to humans, it is expected that prions would accumulate in SLOs. Therefore, histopathological examination and biochemical analysis of PrP^Sc^ in SLOs should be conducted for all young individuals succumbed to CJD, as well as older individuals presenting with atypical clinical or neuropathological features. Additionally, transmission studies in animal models could provide further insights into distinguishing between sCJD and zoonotic forms of CJD.

Lymphotropic prion strains, such as BSE, acquired through cross-species transmission, can persist stably and silently in SLOs for extended periods without neuroinvasion (Peden et al., 2010; Bishop et al., 2013). In fact, SLOs serve as silent reservoirs of prion infection, where prions may remain undetected while posing a potential risk of transmission (Hilton et al., 2004a; Peden et al., 2004; Wroe et al., 2006; Peden et al., 2010; Bishop et al., 2013; Gill et al., 2013). Screening human lymphoreticular tissues in regions with a long history of CWD could provide valuable insights into whether CWD prions are silently harbored within the human population.

In conclusion, the expanding scale of human exposure to the growing CWD epidemic necessitates urgent discussions on safeguarding public health. The implementation of lymphoid tissue autopsies could aid in differentiating between sCJD from CJD acquired via transmission. Furthermore, improved risk assessment for CWD transmission to humans could be achieved by analyzing PrP^Sc^ accumulation in both the spleen and brain following ic inoculation of humanized mice along with the use of humanized mouse models with the 129MV genotype.
